# Secondary Site Ligand for Integrin *α*V*β*3 Enables Targeted mRNA Delivery

**DOI:** 10.1002/anie.1554405

**Published:** 2026-06-18

**Authors:** Sebastian Bayer, María García‐García, Raffaele Senatore, Maria Stratoudaki, Alina Markhof, Angelika Berger‐Becvar, Anna Sophia Parianou, Christoph Rademacher

**Affiliations:** ^1^ Department of Pharmaceutical Sciences University of Vienna Vienna Austria; ^2^ Vienna Doctoral School of Pharmaceutical Nutritional and Sport Sciences University of Vienna Austria; ^3^ Department of Microbiology Immunology and Genetics Max F. Perutz Labs Vienna Austria

**Keywords:** angiogenesis, fragment‐based drug discovery (FBDD), integrin *α*V*β*3, lipid nanoparticles (LNPs), ligand‐directed delivery, multivalency and avidity, mRNA delivery, non‐RGD integrin ligands, superselectivity, vascular targeting

## Abstract

Poor perfusion and abnormal vasculature constrain direct drug delivery to solid tumors. Hence, targeting neighboring tumor endothelial cells via the upregulated marker integrin *α*V*β*3 is a promising strategy. Orthosteric arginine–glycine–aspartate (RGD) ligands of *α*V*β*3 achieve high affinities but suffer from cross‐reactivity. Alternatively, selective targeting of *α*V*β*3 could potentially be achieved via low‐affinity ligands, displayed multivalently on nanoparticles to leverage avidity. To avoid orthosteric site competition, we performed a fragment screening under RGD saturation. Structure–activity relationship (SAR) analysis of the initial hit revealed its binding motif, a 4‐methylpyrimidine‐2‐amine core and a conjugation‐tolerant position for linker attachment. Multivalent display of the lead compound on liposomes and lipid nanoparticles (LNPs) led to time‐, dose‐, and valency‐dependent uptake in *α*V*β*3‐expressing model cells and in primary human umbilical vein endothelial cells (pHUVECs). In contrast to RGD‐decorated NPs, fragment‐targeted NPs show superselective behavior (*α*_κ_≈3), that is, a sharp valency‐dependent threshold, enabling selective targeting of cells overexpressing *α*V*β*3. Crucially, mRNA delivered by targeted LNPs translated into functional protein expression six‐fold over control. To our knowledge, these findings present the first non‐orthosteric, exogenous small‐molecule ligands for *α*V*β*3 and outline their application as a multivalent, superselective delivery platform.

## Graphical abstract

Non‐orthosteric, low‐affinity ligands for integrin *α*V*β*3, identified by fragment screening, achieve superselective nanoparticle targeting (*α*_κ_ ≈ 3) of *α*V*β*3‐overexpressing endothelial cells. Unlike traditional arginine–glycine–aspartate decorated particles, these fragment‐functionalized LNPs discriminate cells by receptor density and deliver mRNA to primary human umbilical vein endothelial cells with six‐fold enhanced translation.

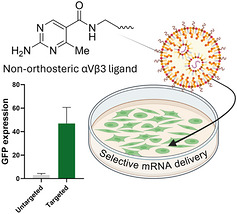

## 1 Introduction

Integrins are heterodimeric transmembrane adhesion receptors that couple the extracellular matrix to the actin cytoskeleton inside the cell and enable bidirectional signaling that governs cell survival, migration and angiogenesis [1, 2]. Among them, integrin *α*V*β*3 is tightly linked to endothelial remodeling and the neovasculature of solid tumors, making it an attractive receptor for targeted anticancer therapy [3, 4, 5]. Conventionally, drug discovery for integrin *α*V*β*3 has focused on arginine–glycine–aspartate (RGD)‐mimetic motifs that engage a conserved canonical ligand‐binding pocket shared across multiple RGD‐recognizing integrins (e.g., *α*V*β*3/*β*5/*β*6/*β*8) [6, 7]. While this strategy has resulted in high‐affinity antagonists and clinical candidates, it also revealed class‐wide limitations, including cross‐reactivity within the RGD‐recognizing subfamily, unexpected conformation‐dependent pharmacology, and, in some cases, agonist‐like conformational shifts that complicate clinical translation [8, 9, 10]. A classic example is the dual *αV* *β*3/*αV* *β*5 inhibitor cilengitide, the only *α*V‐integrin targeting agent to have entered phase III clinical trial, which, however, failed to demonstrate an overall survival benefit in the CENTRIC trial [11]. Low levels of cilengitide even induce tumor angiogenesis by altering integrin αVβ3 trafficking and thereby promoting endothelial cell migration to VEGF, highlighting the complex biology of this receptor family [10].

Given the limited clinical success of integrin *α*V*β*3 antagonism, emphasis has shifted from receptor inhibition to receptor‐addressed delivery [9, 12]. Nanoparticles, such as liposomes and lipid nanoparticles (LNPs), can display various targeting ligands while carrying diverse payloads (small molecules, proteins, nucleic acids). RGD‐decorated formulations leverage rapid endocytosis of integrin *α*V*β*3 upon ligand binding, and several groups have reported improved mRNA translation relative to non‐targeted controls [13, 14]. However, these systems fail to improve biodistribution; they do not significantly increase tumor accumulation in vivo. They, like other active targeted NP systems, are hindered by heterogeneous receptor expression, protein corona formation, off‐target uptake and poor endosomal escape [15, 16, 17, 18]. One reason for these limitations of targeted nanocarriers is that the prevailing, antibody‐inspired paradigm equates specificity with a single high‐affinity interaction and presumes the presence of a uniquely presented target, for example, a receptor only expressed on tumor cells, whereas in reality, most targets are found elsewhere at lower abundance, giving rise to off‐target effects.

In contrast, surface discrimination in biological systems often occurs via a different strategy; glycan‐protein interactions rely on multiple weak binding events [19, 20]. When arrayed multivalently, their avidity overcomes low monovalent affinity and can create “superselective” binding, a steep, density‐dependent off/on switch that enables discrimination of high‐density targets, while sparing low‐level backgrounds. In theoretical models, this behavior is quantified by the selectivity parameter

$$\alpha =\frac{d\ln\;\theta }{d\ln\;n_{R}}$$

where ***θ*** is the fraction of bound particles and ***n***_***R***_ the receptor surface density; values of **α** > 1 define the superselective regime [21]. Crucially, if individual ligand–receptor bonds are too strong, particles bind even to sparsely decorated surfaces, and this density dependence is lost, whereas weak monovalent interactions keep binding off at low receptor density and sharpen the multivalent threshold. Therefore, weaker ligands can be advantageous when displayed multivalently. Based on this, we and others argue that selectivity in drug delivery to cells that overexpress target receptors compared to other cell types can be achieved with low‐affinity ligands, provided they reach a suitable level of target specificity [21, 22, 23]. Guided by this logic, we pursue non‐RGD, secondary‐site small molecules as monovalent binders that become potent and density‐discriminating upon multivalent presentation, while avoiding competition at the conserved RGD pocket and its intrinsic cross‐reactivity.

To test this hypothesis, we biased a fragment screening via RGD blockade to identify a conjugation‐ready chemotype, arrayed it on lipid‐based nanoparticles, and evaluated uptake and payload delivery in receptor‐overexpressing model cell lines and primary human umbilical vein endothelial cells (pHUVECs). The resulting nanoparticles exhibited receptor‐density–dependent superselectivity, and rapid endocytosis and secondary‐site engagement could be confirmed. Importantly, *α*V*β*3‐targeted LNPs mediated robust cytosolic mRNA delivery and translation relative to non‐targeted controls. These findings introduce a non‐orthosteric, superselective targeting strategy for *α*V*β*3 that supports selective nucleic acid delivery to tumor endothelial models and suggests broader applicability to other overexpressed adhesion receptors.

## 2 Results and Discussion

### 2.1 Fragment Screening for Non‐RGD Binding Sites

**FIGURE 1:**
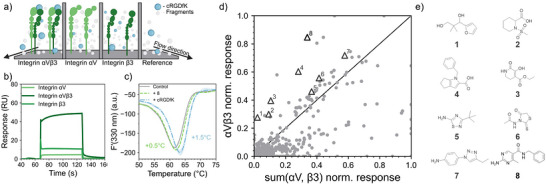
Fragment screening under RGD blockade identifies *α*V*β*3‐selective secondary‐site hits. (a) Schematic representation of the GCI screening setup. Immobilized integrin *α*V*β*3 heterodimer, *α*V and *β*3 monomers, and a reference channel are probed in parallel against a fragment library. The assay is performed in the continuous presence of saturating concentrations of cRGDfK to block the primary binding site. (b) Representative GCI sensorgrams of hit **8** against *α*V*β*3 (dark green), *α*V (light green), and *β*3 (green). The data show selective binding to the heterodimer with negligible response for the isolated monomers. (c) TSA melting curves of *α*V*β*3 alone (grey), with hit **8** (green), or with the positive control cRGDfK (blue). The shift to higher temperatures indicates stabilization of the protein by ligand binding. (d) Scatter plot of fragment responses normalized for molecular weight (MW), plotted as normalized signal against *α*V*β*3 versus the sum of monomer signals (*α*V+*β*3). Orthogonally confirmed *α*V*β*3‐specific hits are highlighted as black triangles. (e) Chemical structures of the identified secondary‐site hits.

To identify hits from our in‐house fragment library against non‐canonical RGD sites of integrin *α*v*β*3, we used grating‐coupled interferometry (GCI) [24]. For this label‐free method, we immobilized three receptor constructs plus an unlabeled control channel on the chip surface and performed parallel screening of the integrin *α*V*β*3 heterodimer and the two monomers *α*V and *β*3, respectively, under cRGDfK saturating conditions (Figure 1a). Out of 788 compounds tested, 7% (56) showed a normalized response of over 0.2 against integrin *α*V*β*3 and were classified as hits. Of these, 22 hits had a preference for integrin *α*V*β*3 over the monomers *α*V and *β*3 (Figure 1d). Next, we tested these hits in an orthogonal thermal shift assay (TSA) and validated eight compounds showing a significant increase in melting temperature (≥0.5°C) (Figure 1c–e). Out of those, compounds **1**, **3**, **4**, and **8** showed at least a two‐fold higher response in the original GCI assay against the dimer over the monomers. From those, compound **8** (2‐amino‐*N*‐benzyl‐4‐methylpyrimidine‐5‐carboxamide) was selected for further studies, based on synthetic tractability and potential for chemical elaboration.

### 2.2 Structure–Activity Relationship (SAR) of Compound **8** Reveals Binding Motif and Conjugation Site for Multivalent Display on Liposomes

**FIGURE 2:**
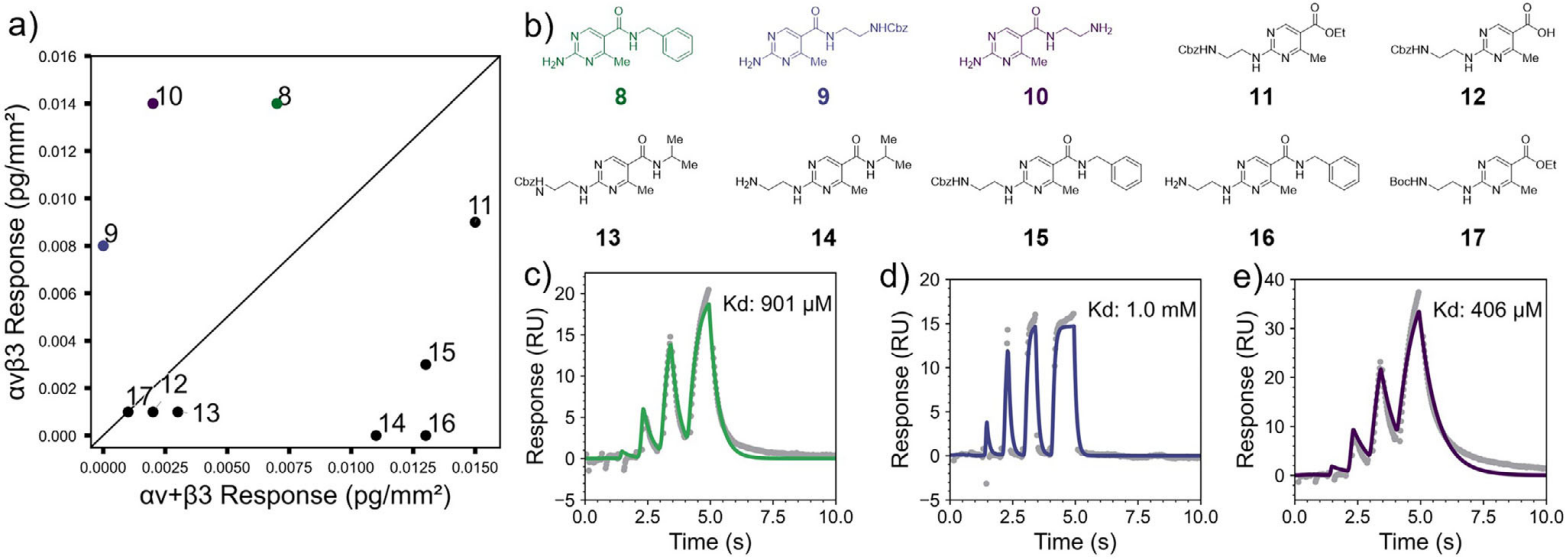
SAR study of hit **8** identifies the binding motif and a conjugation vector. (a) GCI responses of **8** and analogues 9–17 were measured under the same set‐up as in Figure 1. (b) Chemical structures of the parent hit **8** (green) and analogues **9** (blue) and **10** (purple), which retain *α*V*β*3 potency and selectivity, alongside inactive or non‐selective derivatives **11**–**17**. (c–e) waveRAPID GCI sensorgrams for the *α*V*β*3 selective binders **8** (c), **9** (d), and **10** (e). Experimental data are shown as grey points, with kinetic fits in the corresponding compound color. KD values are indicated, showing micromolar to millimolar affinity.

**SCHEME 1:**
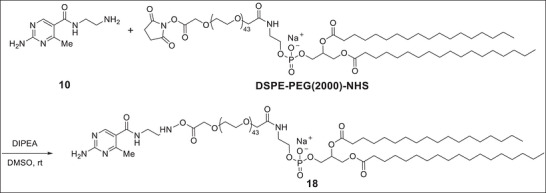
Amine coupling of 10 to DSPE‐PEG2000‐NHS to afford compound **18**.

For advancing the weak‐binding hit **8** into a targeting ligand for active display on nanoparticles, we set out to identify conjugation sites that would leave the binding epitope intact. Thus, nine analogues (**9**‒**17**) were synthesized and tested in GCI under c(RGDfK) saturating conditions in the same binding experiment and additionally via waveRAPID to determine affinities (Figure 2). **8** itself showed a high µM Kd against *α*V*β*3 (Kd *_α_*_V_ *_β_*_3_ = 901 µM), in line with expectations of an initial fragment hit (Figure 2c). All substitutions on the primary amine of the 4‐methylpyrimidine‐2‐amine core led to a decrease in response for the *α*V*β*3 heterodimer, and bulky, 3D substituents even abolished monomer binding (Figure 2a,b). In contrast, exchanging the benzyl moiety for a primary amine (10, Kd*_α_*_V_ *_β_*_3_ = 406 µM) increased the selectivity toward the heterodimer by three‐fold (Figure 2a–e). Extending the connecting substituted alkyl chain was tolerated (**9**, Kd*_α_*_V_ *_β_*_3_ = 1012 µM) (Figure 2d). From this, we concluded that the 4‐methylpyrimidine‐2‐amine is important for the specific interaction, while the benzyl group can be substituted and used as a conjugation site. Therefore, we chose to continue with **10**, which showed the highest selectivity for the dimer and has a primary amine for coupling to DSPE‐PEG(2000)‐NHS, resulting in **18** (Scheme 1). Future investigation could explore linker optimization such as spacer length or rigidity. Next, liposomes with up to 2 mol% **18** in formulations containing DSPC:cholesterol:DSPE‐PEG_2000_ (ratios of 57,5:37,5:5) were prepared. These formulations are of similar composition to approved DOXIL and include Alexa Fluor 647 for cellular tracking [25]. The resulting liposomal formulations showed homogeneous particle size distributions averaging 89.2 ± 4.7 nm with polydispersity indices (PDIs) below 0.22 (Figure S1a,b).

### 2.3 Selective Uptake of 18 Displaying Liposomes by Integrin *α*V*β*3‐Expressing HEK293T Cells

**FIGURE 3:**
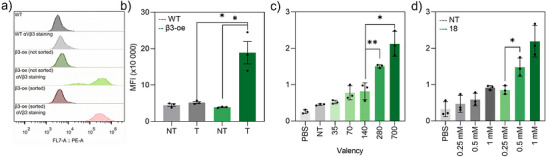
*α*V*β*3‐dependent uptake of **18**‐decorated liposomes in engineered *αVβ*3‐expressing HEK293T cells. (a) Flow cytometry characterization of surface integrin expression. Histograms compare HEK293T wild‐type (WT, grey) and *β*3 ‐transfected cells (green/red). Sorting of the transfected population yields a cell line with high surface presentation of integrin *α*V*β*3 heterodimer (red). (b) Quantification of cellular uptake by mean fluorescence intensity (MFI). WT and *β*3‐overexpressing (*β*3‐oe) cells were incubated with non‐targeted (NT, grey) or **18**‐decorated (T, green) liposomes. Significant uptake is observed only for the targeted formulation in *β*3‐oe cells. (c) Effect of ligand valency on internalization. *β*3‐oe cells treated with liposomes displaying increasing valencies of **18** (35–700 ligands per liposome) show a valency‐dependent increase in MFI. (d) Dose‐response analysis. Uptake of NT (grey) and **18**‐decorated (green) liposomes at increasing lipid concentrations (0.25–1.0 mM) shows increasing MFI values for targeted liposomes. Data represent mean ± SD (*n* = 3). Statistical significance was assessed by two‐way ANOVA with Tukey's multiple comparisons test (b) and Dunnett's multiple comparisons test against the NT control (c), or two‐way ANOVA with Sidak's multiple comparisons test (d). *: *p* < 0.05, **: *p* < 0.01.

We chose HEK293T cells to assess selectivity for Integrin *α*V*β*3 because these cells endogenously express the *α*V monomer and, after engineered expression of *β*3, form cell‐surface‐exposed *α*V*β*3 (Figure 3a) [26, 27]. Moreover, the endogenous *α*V expression comes with a large palette of other integrins being cell surface exposed, and consequently, HEK293T WT cells serve as an excellent control to quantify potential off‐target interactions such as *α*V*β*1 [26, 27]. Therefore, we studied liposome uptake of **18**‐targeted versus non‐targeted liposomes in wild‐type (WT) and *α*V*β*3‐expressing HEK293T cells. Targeted liposomes were robustly internalized by *α*V*β*3‐expressing HEK293T cells, far exceeding non‐targeted controls, whereas WT cells showed no detectable uptake of targeted liposomes over controls (Figure 3b). Binding of targeted liposomes was observed to be valency and concentration‐dependent (Figure 3c,d). This confirms that **18**‐targeted liposomes show excellent binding and uptake by *α*V*β*3‐expressing cells and, at the same time, also underscores their high selectivity.

### 2.4 Time and Concentration‐Dependent Uptake of Targeted Liposomes in Primary Cells

**FIGURE 4:**
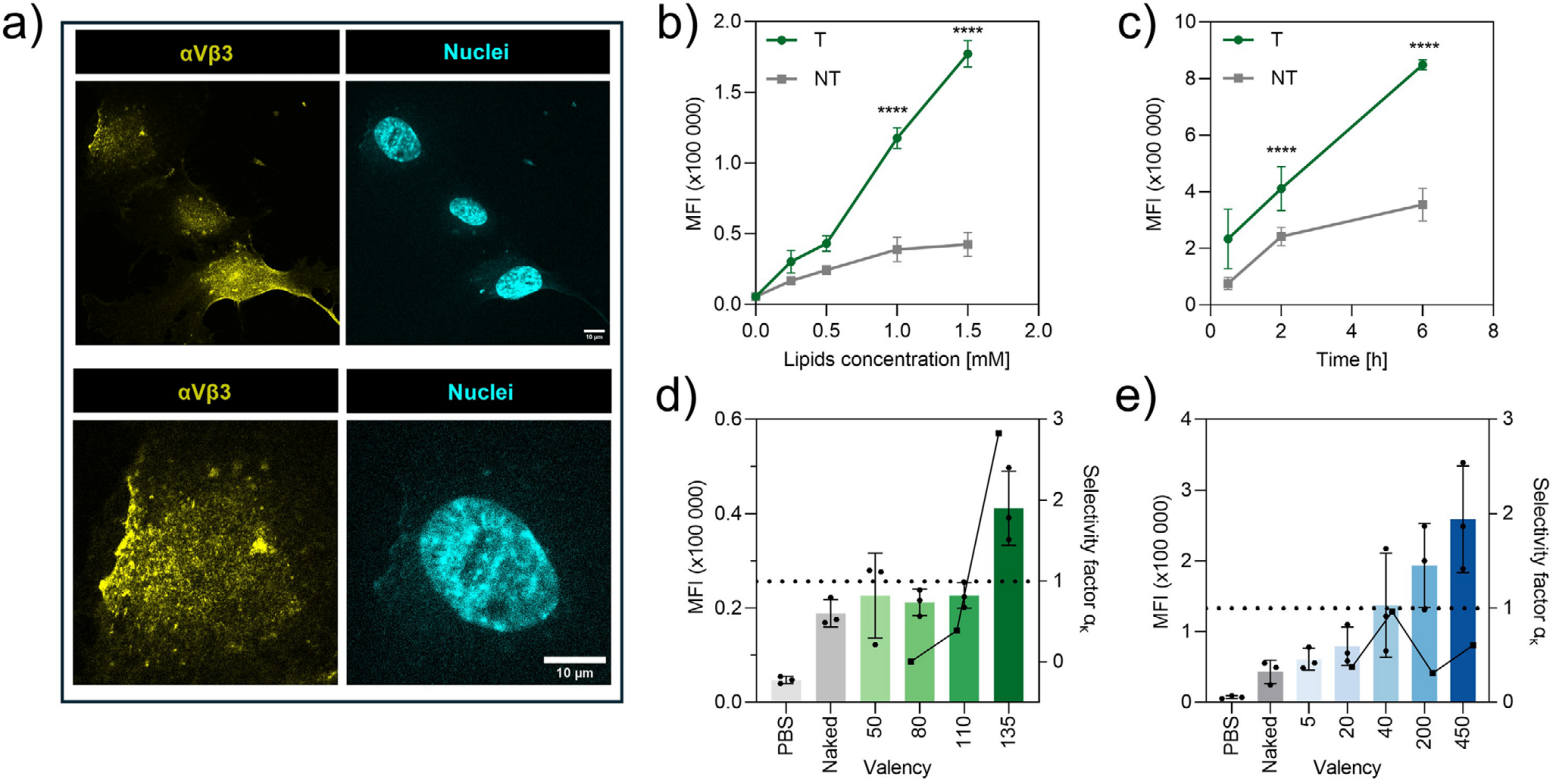
**18**‐Decorated liposomes bind to pHUVECs, displaying superselective behavior. (a) Confocal immunofluorescence microscopy of pHUVECs. Staining for integrin *α*V*β*3 (yellow) and nuclei (cyan) confirmed high receptor expression. Bottom images are an ROI of the upper ones. Scale bar: 10 µm. (b) Concentration‐dependent uptake. pHUVECs were incubated with non‐targeted (NT, grey squares) or **18**‐decorated (green circles) liposomes (0–1.5 mM lipid concentration). Targeted formulation showed up to ∼eight‐fold higher MFI. (c) Time‐course of cellular uptake. Cells treated with NT or **18**‐decorated liposomes (T) (0.5 mM) were analyzed at 30 min, 2 h, and 6 h, confirming progressive accumulation in line with rapid receptor cycling. (d, e) Evaluation of binding superselectivity. MFI (bars, left axis) and the local selectivity factor *α* (black line, right axis) are plotted against ligand valency for **18**‐liposomes (d) and cRGDfK‐liposomes (e). **18**‐targeted liposomes show negligible binding below a density threshold, followed by a sharp increase with a maximum selectivity factor *α*≈2.9, characteristic of superselective behavior. In contrast, high‐affinity cRGDfK liposomes show gradual binding increases (*α* < 1) across the valency range. The dotted line at *α* = 1 marks the threshold for superselectivity. Data represent mean ± SD (*n* = 3). Statistical significance was assessed by two‐way ANOVA with Sidak's multiple comparisons test (b, c), *: *p* < 0.05,***: *p* < 0.001,****: *p* < 0.0001.

To evaluate **18**‐targeted liposomes in a standard human model of angiogenic endothelium, we used pHUVECs. pHUVECs express integrin *α*V*β*3 at a high level on their surface (Figure 4a). Targeted liposomes bound to pHUVECs in a concentration‐dependent manner up to eight‐fold higher than the non‐targeted control (Figure 4b). Since Integrin *α*V*β*3 undergoes rapid endocytosis and receptor recycling [28] (Figure S3), *α*V*β*3‐targeted liposomes should also be taken up in a time‐dependent manner, without receptor fatigue. Indeed, uptake of targeted particles increased over multiple time points up to 6 h, showing an increasing fold change in comparison to control (Figure 4c). These findings show time and concentration‐dependent internalization of targeted liposomes in a primary endothelial cell model.

### 2.5 18‐Targeted Liposomes Exhibit Valency‐Dependent Binding Consistent With Superselectivity

Next, we asked whether **18**‐targeted liposomes show density‐dependent binding consistent with superselective behavior toward *α*V*β*3‐expressing pHUVECs. We followed the liposomal binding to pHUVECs while systematically varying the valency (***κ***) of 18 on the liposome surface from 50 to 135 outward facing ligand molecules per liposome. Analogous to ***α*** by Martinez–Veracoechea and Frenkel [21], we defined the valency‐based selectivity exponent

$$\;\alpha_{\kappa }=\frac{d\ln\;\theta }{d\ln\;\kappa }$$

With the number of bound particles (***θ***)* *approximated experimentally by the MFI. Uptake of **18**‐decorated liposomes remained negligible up to ***κ*** ≈ 110, above which a sharp increase of ***θ*** was observed, consistent with a threshold‐like superselective response (Figure 4d). The local ***α***_***κ***_ **at this onset was ≈2.9, indicating a steep dependence on ligand valency. By contrast, cRGDfK‐liposomes showed a more gradual, approximately linear increase in binding to the pHUVECs, with ***α***_***κ***_ remaining <1, consistent with non‐superselective behavior (Figure 4e). This is consistent with numerous reports that RGD‐decorated liposomes and nanoparticles exhibit broad, non‐specific uptake rather than selectively targeting *α*V*β*3 overexpressing surfaces [29, 30, 31]. In contrast, the weak monovalent interaction of **18** avoids binding at low valency yet supports a sharp multivalent threshold at high ligand valency *κ*. These data support the notion that low‐affinity ligands are better suited than classical RGD motifs to exploit superselective binding for receptor‐density discrimination in targeted delivery.

### 2.6 Competition Confirms Non‐RGD, Secondary Site Targeting

**FIGURE 5:**
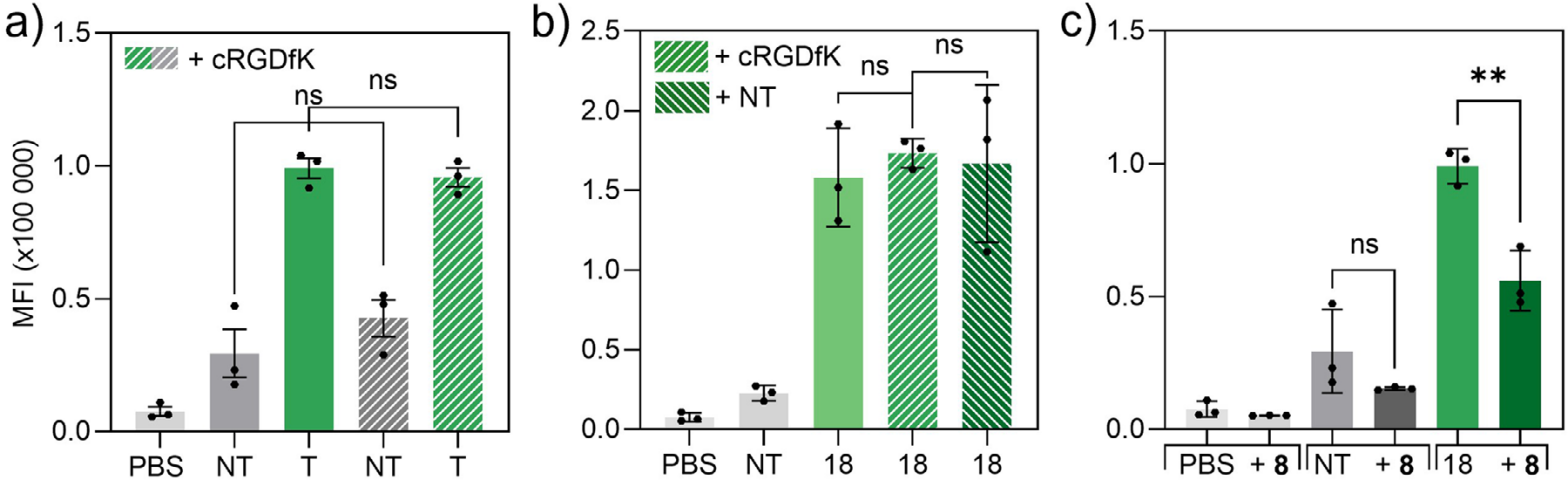
Competition experiments confirm secondary‐site engagement of *α*V*β*3 by **18**‐decorated liposomes in pHUVECs. (a) Orthosteric site competition with a free competitor. Uptake of non‐targeted (NT, grey) or **18**‐decorated (T, green) liposomes following pre‐incubation with excess soluble cRGDfK peptide (500 µM). The lack of inhibition indicates the RGD binding pocket is not required for targeting. (b) Liposomal competition. Uptake of **18**‐decorated liposomes is unaffected by the co‐administration of high‐affinity cRGDfK‐decorated liposomes, consistent with the engagement of distinct, non‐overlapping binding sites. (c) Secondary‐site blockade via free **8**. Co‐incubation with the free monovalent fragment **8** significantly reduces the uptake of **18**‐decorated liposomes, confirming that the multivalent liposomal ligand and the small molecule address the same non‐orthosteric pocket. Data represent mean ± SD (*n* = 3). Statistical significance was assessed by unpaired two‐tailed *t*‐test (a, b) and one‐way ANOVA with Tukey's multiple comparisons test (c). **: *p* < 0.01, ns: not significant.

To confirm non‐orthosteric binding, we performed competition experiments using the primary‐site ligand cRGDfK, both in its free form (Figure 5a) and as a liposome‐bound form (Figures 5b and S5a,b). No effect on **18**‐based liposome uptake in pHUVECs was observed. On the other hand, using **8** as competitor significantly reduced **18**‐based targeted liposome internalization into pHUVEC cells (Figure 5c). Together, the lack of competition by cRGDfK and the inhibition by 8 demonstrate that compound **18**‐driven uptake does not engage the orthosteric RGD pocket but instead relies on a non‐orthosteric (secondary) site on *α*V*β*3.

### 2.7 Targeted Delivery and Translation of mRNA in Primary Cells

**FIGURE 6:**
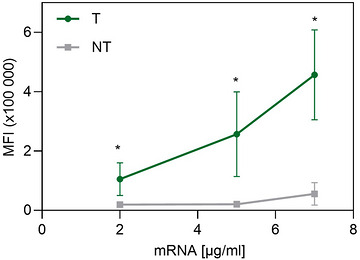
*α*V*β*3‐targeted LNPs mediate functional cytosolic delivery and translation of eGFP mRNA in pHUVECs. Cells were treated with non‐targeted (NT, grey) or **18**‐decorated (green) LNPs encapsulating 2, 5 or 7 µg/ml eGFP mRNA. The targeted LNPs induce markedly higher GFP levels than non‐targeted controls (up to ∼six‐fold enhancement. Data represent mean ± SD (*n* = 4). Statistical significance was assessed by two‐way ANOVA with Sidak's multiple comparisons test *: *p* < 0.05.

Having established receptor‐selective delivery of **18**‐targeted liposomes to integrin *α*V*β*3‐expressing cells (pHUVEC and engineered *α*V*β*3 expressing HEK293T cells), we next investigated whether this receptor–ligand interaction enabled cytosolic delivery of a biologically active cargo. To address endosomal escape and cytosolic delivery, we formulated ionizable lipid‐containing formulations designed for nucleic acid delivery, henceforth called LNPs, encapsulating uracil‐modified mRNA encoding eGFP. For this, LNPs containing DSPC, cholesterol, the ionizable lipid ALC‐0315, and DSPE‐PEG_2000_ (10:38.37:50:1.62), including 0.12 mol% DSPE‐PEG_2000_‐AlexaFluor647 for detection, were prepared. Targeted particles included 0.45 mol% compound **18** (corresponding to a valency of ∼450) and 1.05% DSPE‐PEG_2000_ (compared to non‐targeted with 1.5 mol% DSPE‐PEG_2000_) (Figure S8a,b) with high mRNA encapsulation efficiencies (Figure S8c). Dose‐dependent eGFP expression by pHUVEC treated with **18**‐targeted LNPs was detected after 24 h (Figure 6). These data confirm that compound **18**‐mediated targeting of integrin *α*V*β*3 not only promotes cellular uptake of the nanoparticles but also facilitates effective cytosolic delivery and translation of mRNA cargo in pHUVEC cells, which, to our knowledge, has not been realized with RGD‐targeted delivery thus far.

Receptor‐specific binding and uptake alone are not sufficient for functional delivery: productive expression requires that the internalized particles release their mRNA into the cytosol by escaping the endosomal pathway, a step that is poorly understood and remains widely regarded as the dominant bottleneck for non‐viral systems, including LNPs [18]. In parallel to the outlined work, a second ligand series (derived from compound **2**) yielded similar cellular uptake in HEK293T and pHUVEC assays compared to the compound‐**8**/**18** series, yet failed to increase GFP expression over non‐targeted controls (Figure S8a–e). Given similar monovalent affinities for both hits (Kd *_α_*_v_ *_β_*_3_: **8** = 901 µM, vs. **2** = 626 µM, Figures 2c and S8f), we can only speculate as to the reason for this difference in behavior: ligand identity can bias integrin routing and residence time (recycling vs. late endosomes/lysosomes), modulate nanoscale clustering/PEG architecture that governs escape competence, reshape the protein corona (e.g., ApoE) and affect local pH exposure‐potentially mismatching the ionizable‐lipid *p*K_a_ window for membrane disruption [32, 33, 34, 35]. This renders the **8**/**18** series particularly valuable: unlike other ligands with comparable binding, it reliably converts *α*V*β*3 recognition into functional mRNA delivery with up to six‐fold GFP translation over control under matched conditions (Figure 6).

## 3 Conclusion

We identified the first exogenous, non‐orthosteric small‐molecule ligands for integrin *α*V*β*3. Multivalent display of the compound **18** yields ligand valency–dependent superselectivity (**α**_***κ***_= 2.9 at binding onset) and exploits rapid *α*V*β*3 endocytosis to drive uptake in *α*V*β*3 engineered cells and primary human endothelial cells. Engagement of a secondary binding site is demonstrated in biophysical assays as well as in cells in vitro. Furthermore, targeting translates into up to six‐fold higher messenger RNA translation versus non‐targeted controls. Beyond the novel chemotypes and their application, these results establish a rapid fragment‐to‐multivalency workflow for cell‐surface receptors that avoids liabilities of high‐affinity, orthosteric ligands and can be tuned by ligand density to discriminate high‐ from low‐expressing cells depending on context.

## Author Contributions

**Sebastian Bayer**: conceptualization, methodology, investigation, writing – original draft, writing – review and editing. **María García‐García**: conceptualization, methodology, investigation, writing – review and editing. **Raffaele Senatore**: conceptualization, methodology, investigation. **Maria Stratoudaki**: investigation. **Alina Markhof**: investigation. **Angelika Berger‐Becvar**: investigation. **Anna Sophia Parianou**: investigation. **Christoph Rademacher**: conceptualization, methodology, writing – original draft, writing – review and editing, supervision, funding acquisition. SB conducted GCI and TSA, RS conducted synthetic chemistry of SAR and DSPE‐PEG coupling, MGG, MS and ABB conducted liposomal and LNP formulations, MGG, ASP, MS and AM conducted cell experiments (HEK293T and pHUVECs).

## Conflicts of Interest

The authors declare no conflicts of interest.

## Supporting information




**Supporting File**: anie72921‐sup‐0001‐SuppMat.docx.

## Data Availability

The data that support the findings of this study are available from the corresponding author upon reasonable request.
