# Supplementary material for: Secondary Site Ligand for Integrin αVβ3 Enables Targeted mRNA Delivery
Source: Angew Chem Int Ed Engl. 2026 Jun 18;65(34):e1554405. doi: 10.1002/anie.1554405 (PMC13480715; doi:10.1002/anie.1554405)
Supplement: Supplementary file 1 [file ANIE-65-e1554405-s001.docx]

Secondary site ligand for Integrin αVβ3 Enables Targeted mRNA Delivery

Supporting Information

María García-García ^[a]^ , Sebastian Bayer ^[a, b]^, Raffaele Senatore ^[a]^, Maria Stratoudaki ^[a]^,

Alina Markhof ^[a,b]^, Angelika Berger-Becvar ^[a]^, Anna Sophia Parianou ^[a,b]^, Christoph Rademacher ^[a,c] *^

[a] Department of Pharmaceutical Sciences, University of Vienna, Josef-Holaubek-Platz

2, 1090 Vienna, Austria

[b] Vienna Doctoral School of Pharmaceutical, Nutritional and Sport Sciences, University

of Vienna, Austria

[c] Department of Microbiology, Immunology and Genetics, Max F. Perutz Labs, Biocenter

5, 1030 Vienna, Austria

Email: *Christoph.Rademacher@univie.ac.at

Table of contents

[Experimental Procedures	2](#_Toc216443769)

[Biophysical assays and screening	3](#_Toc216443770)

[Recombinant Integrin αvβ3, Integrin αv and Integrin β3	3](#_Toc216443771)

[Fragment library	3](#_Toc216443772)

[Fragment stock preparation	3](#_Toc216443773)

[GCI measurements and screening	3](#_Toc216443774)

[TSA measurements	4](#_Toc216443775)

[GCI measurements of SAR compounds	4](#_Toc216443776)

[Nanoparticle formulation and cell culture experiments	4](#_Toc216443777)

[Cell culture, reagents and plasmids	4](#_Toc216443778)

[Immunofluorescence and microscopy	5](#_Toc216443779)

[Immunostaining and FACS	5](#_Toc216443780)

[Endocytosis Assay	5](#_Toc216443781)

[Nanoparticle formulation	5](#_Toc216443782)

[Nanoparticle characterization with Dynamic Light Scattering	6](#_Toc216443783)

[Nanoparticle binding/uptake experiments	6](#_Toc216443784)

[Western blotting	6](#_Toc216443785)

[Synthetic chemistry	7](#_Toc216443786)

[Instrumentation and general analytical methods.	7](#_Toc216443787)

[Synthesis, characterization and spectral data of compounds.	8](#_Toc216443788)

[Synthesis schemes	14](#_Toc216443789)

[1H and 13C NMR spectra	15](#_Toc216443790)

[Supporting figures	30](#_Toc216443791)

[Figure S1. Size characterization of compound 18–decorated liposomes.	30](#_Toc216443792)

[Figure S2. Rapid endocytosis of integrin αVβ3 on pHUVECs	31](#_Toc216443793)

[Figure S3. Donor-dependent integrin αVβ3 expression in pHUVECs	31](#_Toc216443794)

[Figure S4. Size characterization of cRGDfK–decorated liposomes.	32](#_Toc216443795)

[Figure S5. Soluble cRGDfK competes with cRGDfK–decorated liposome binding.	33](#_Toc216443796)

[Figure S6. Size characterization of compound 18–decorated LNPs.	34](#_Toc216443797)

[Figure S7. Compound 2/25 series with similar biophysical affinity and targeting performance in pHUVECs fails to increase mRNA translation.	35](#_Toc216443798)

[Supporting references	36](#_Toc216443799)

# Experimental Procedures

Unless stated otherwise, all reagents and buffers were obtained from Sigma Aldrich or Carl Roth.

## Biophysical assays and screening

### Recombinant Integrin αvβ3, Integrin αv and Integrin β3

All proteins used for biophysical studies were bought from commercial vendors. Integrin αvβ3 from SinoBiological (Cat.Nr.: CT098-H08H), Integrin αv from Origene (Cat.Nr.:TP762381) and Integrin β3 from Aviva systems biology (Cat.Nr.: OPCD04595). Proteins were received under freeze dried conditions. Before performing biophysical assays, proteins were taken up in HBS (25 mM HEPES, pH 7.4, 150 mM sodium chloride) and dialysed in a Slide-A-Lyzer™ MINI Dialysis Device, 7K MWCO (ThermoFisher) against 5l HBS over night at 4°C to remove buffer additives.

### Fragment library

Fragments were selected from a pool of commercially available compounds from different manufacturers guaranteeing purity >95% (Sigma-Aldrich, St. Louis, MO, USA; KeyOrganics, Camelford, UK; ACB Blocks, Toronto, ON, Canada; Santa Cruz Biotechnology, Santa Cruz, CA, USA; Vistas-MLab, Moscow, Russia; LifeChemicals, Kyiv, Ukraine; Alfa Aesar, Ward Hill, MA, USA; TCI, Tokyo, Japan; Apollo Scientific, Stockport, UK; Analyticon, Potsdam, Germany; ChemBridge, San Diego, CA, USA; ChemDiv, San Diego, CA, USA; Enamine, Kyiv, Ukraine; Otava Chemicals, Ontario, Canada; Princeton BioMolecular Research, Princeton, NJ, USA) as well as academic laboratories (Gaich Laboratories, University of Konstanz, Germany; AG Kaufmann, TU Clausthal, Germany; McQuade Research Group, Florida State University, Tallahassee, FL, USA; Nazaré group, Leibniz-Institut für Molekulare Pharmakologie, Berlin, Germany; Werz Research Group, TU Braunschweig, Germany) using chemoinformatic tools as implemented in MOE, Chem Axon, and KNIME as described earlier.^[1]^ Briefly, compounds with <23 non-hydrogen atoms were filtered for rule-of-three, PAINS, and reactives and selected for chemical, scaffold, and shape diversity.

### Fragment stock preparation

Fragment stocks were performed as described earlier.^[1]^ Briefly, fragments were dissolved at 100 mM in d6DMSO and controlled for solubility, stability, and identity at 1 mM in aqueous buffer utilizing 1H and, if applicable, 19F NMR.

### GCI measurements and screening

All GCI experiments were performed on a WAVEsystem (Malvern Panalytical, Malvern UK) using HBS (25 mM HEPES, pH 7.4, 150 mM sodium chloride, 0.0025% Tween-20) as running buffer at 25°C. For this study, Pre-immobilized NTA, thick PC hydrogel (PCH-NTA) sensor chips, which feature 4 channels were used to covalently couple the HIS-tagged proteins via N-hydroxysuccinimide (NHS) / 1-ethyl-3-(3-dimethylaminopropyl)carbodiimide (EDC) reaction on the sensor chip surface according to manufacturer’s protocols. For the initial screening roughly 10 000 RU, 18 000 RU and 9 000 RU of Integrin avB3, Integrin av and Integrin b3 were immobilised on the sensorchip surface respectively. For the follow up SAR roughly 5000 RU, 8000 RU and 4000 RU of Integrin avB3, Integrin av and Integrin b3 were immobilised on the sensorchip surface respectively. After the protein immobilization, the surface was quenched with 1 M ethanolamine (pH 8.5) for 420 s at 10 μL min-1. The reference flow cell 4 was treated accordingly without immobilizing any protein.

Before measuring sample compounds Cyclo(-RGDfK) (Cas.Nr.: 393781-65-2 from Medchem Express) was measured as positive control using RAPID kinetics, applying the standard settings for Intermediate Binders (45s baseline, 25s association, 300s dissociation) at a concentration of 1µM. Measured kD against the heterodimer avB3 was 7µM. See supplementary figure XX)

Screening against Integrin avB3, as well as the monomers Integrin av and Integrin B3 was performed to identify non-orthosteric hits, selective for the Integrin avB3 heterodimer. For this purpose, the three proteins, as well as the untreated reference channel were screened against 788 fragments that were injected for 60 s at 100 μM in HBS with 0.1% DMSO and 100nM Cyclo(-RGDfK) (Cas.Nr.: 393781-65-2 from Medchem Express) applying a flow rate of 100 μL min-1 and a dissociation time of 60 s according to the standard “screening” settings in the WAVE Control 4.5.17 software.

Data adjustment and blank subtraction was performed in the WAVE Control Creoptix (4.5.17) software. Report plot points exported as .csv files and normalized and plotted in Python (3.1).

### TSA measurements

All TSA experiments were performed on a Prometheus NT48 (Nanotemper technologies, Munich, Germany) in in HBS (25 mM HEPES, pH 7.4, 150 mM sodium chloride, 0.0025% Tween-20).
To orthogonally validate the Integrin αvβ3 hits from GCI screening, the protein was prepared at 1mg/ml (5µM) in HBS with either 1mM of fragment , 300µM Cyclo(-RGDfK) (positive control) or 1% DMSO (negative control). Melting curves were recorded from 20 to 75°C at a heating speed of 1°C/min. Experiments were performed in triplicates. Data was analysed in the PR Stability Analysis Software 1.1 using a two-state fit model. Out of 19 tested hits 8 showed an increase in melting temperature of 0.5°C or higher, in comparison to DMSO control and were thus considered as validated hits.

### GCI measurements of SAR compounds

To perform SAR analysis 7C10 analogues, the compounds were again measured via GCI against αvβ3, as well as the monomers Integrin av and Integrin B3. For this purpose, the the compounds were injected for 60 s at 100 μM in HBS with 0.1% DMSO and 100nM Cyclo(-RGDfK) (Cas.Nr.: 393781-65-2 from Medchem Express) applying a flow rate of 100 μL min-1 and a dissociation time of 60 s according to the standard “screening” settings in the WAVE Control 4.5.17 software.

Data adjustment and blank subtraction was performed in the WAVE Control Creoptix (4.5.17) software. Report plot points exported as .csv files and normalized and plotted in Python (3.1).

## Nanoparticle formulation and cell culture experiments

### Cell culture, reagents and plasmids

HEK293T (ATCC CRL-1573) were grown in DMEM supplemented with 10% fetal bovine serum (Cytiva), 2 mM glutamax (Gibco) and 100 µg/ml penicillin and streptomycin (Gibco). human umbilical vein endothelial cells (HUVEC) from human donors were purchased from SIGMA and LONZA and cultured in EGM-2 medium (Lonza). Gelatin-coating was used for HUVEC cell culture. Cell lines were maintained in a humidified atmosphere at 37 °C and 5% CO_2_. Rabbit-made antibodies: anti-αV/ITGAV/CD51 (H-2, sc-376156, Santa Cruz), anti-β3/ITGB3/CD61 (B-7, sc-46655, Santa Cruz), anti-mouse HRP (A9044, Merck), anti-CD51/CD61 (130-103-744, Miltenyi). Mouse-made antibodies: β-actin (sc-47778, Santa Cruz Biotechnology), anti-rabbit HRP (7074, Cell Signaling). pcDNA3.1-β plasmid was ordered from Addgene (#27289). HEK293t cells were transiently transfected with the vector using lipofectamine (Invitrogen). Transfected cells were selected with G418 antibiotic after reaching cell confluency for 2 weeks. After selection, transfected cells were sorted for integrin αVβ3 positive expression in a Cytoflex SRT (Beckman Coulter).

### Immunofluorescence and microscopy

Images were acquired on a Zeiss LSM 790. ImageJ/Fiji was used to adjust brightness and contrast values. For immunostaining, cells were fixed with 4% paraformaldehyde at 37 °C for 10 min, washed 3 times with PBS, permeabilized with 0.2% Triton X-100 for 5 min, blocked with BSA 2%, incubated with primary antibody for 1h at room temperature, and incubated with secondary antibody (1h, room temperature). Nuclei were counterstained with DAPI.

### Immunostaining and FACS

Unspecific binding was blocked with FcR blocking reagent, human or mouse (Miltenyi) at 4°C for 15 min. Then, primary antibodies were incubated 20 min at 4°C, washed, and secondary antibodies were added (20 min at 4°C). Zombie Violet Fixable Viability dye (Biolegend) was used for live/dead staining. After final PBS wash, cells were fixed with 4% paraformaldehyde solution at 4˚C for 5 min, washed with PBS and flow cytometry data was acquired on a Cytoflex S (Beckman Coulter). Data analysis and visualization was performed using FlowJo software.

All targeting ligands and Alexa Fluor 647 Succinimidyl Ester (AF647, Invitrogen) were conjugated *via* amide coupling to DSPE-PEG_2000_ (Avanti Lipid and NOF Corporation). DSPE-PEG_2000_ Carboxy NHS (3 mg, 0.001 mmol, 1 equiv) and the targeting ligand (10 or 24, 0.006 mmol, 6 eq.) were dissolved in DMSO (500 µl). DIPEA (2.5 µl) was added and the reaction stirred overnight at 40 °C. The reaction mixture was dialyzed three times against 0.1M NaHCO_3_ (1 liter) and three times against water (1 liter) using Slide-A-Lyzer^TM^ cassettes 2K MWCO. After lyophilization, the lipid conjugates were obtained as white solids. The same procedure was followed to couple cRGDfK peptide to DSPE-PEG_2000_, dialyzing the reaction mixture only against water three times.

### Endocytosis Assay

pHUVEC were detached and stained at 4°C following the immunostaining protocol described earlier. Thecells were divided in 5 tubes. Two were left on ice and three were placed in a 37°C incubator. After 10 min, one tube on ice and one tube in the incubator were treated with anti-FITC oligoclonal antibody (Invitrogen) for 20 min to quench CD31/CD61-FITC fluorescence at the plasma membrane. FITC fluorescence was measured in a Cytoflex S (Beckman Coulter) without fixation.

### Nanoparticle formulation

Nanoparticles were prepared by mixing an organic phase and an aqueous phase in a custom-made, S-shape, herringbone microfluidics chip (prepared on Polydimethylsiloxane (PDMS), Wunderlichips GmbH) using a syringe pump (Pump 33 Dual Drive System Syringe Pump, Harvard Apparatus). The flow rate ratio (FRR) of the liposome formulation was set to 1:4 (organic:aqueous phase) , while the FRR of the LNPs formulations to 1:10. The organic phase contained the lipid mixture (5 mM) in 100% ethanol (Sigma), while the aqueous phase consisted of either PBS (Gibco) in the liposomal formulations or 50 mM Sodium Acetate Buffer pH 4.4 (Invitrogen) containing the Uracil-modified mRNA for GFP (Trilink Biotech) in the LNP formulations.

Liposomal formulations were prepared containing 57.5 mol% DSPC (NOF Corporation), 37.5 mol% Cholesterol (Sigma), 0.125 mol% DSPE-PEG_2000_-AF647, 4.875 mol% DSPE-PEG_2000_ (including ligands). The organic phase of LNPs formulations was prepared containing 50 mol% ALC-0315 (Medkoo Biosciences), 38.5 mol% Cholesterol (Sigma), 10 mol% DSPC (NOF Corporation), 0.125 mol% DSPE-PEG_2000_-AF647 and 1.375 mol% DSPE-PEG_2000_/DSPE-PEG_2000_-10.

After mixing in the chip, the product was collected from the outlet and the formulations were dialyzed using 100 kDa Spectrum Dialysis Membrane Tubing (Fisher Scientific) overnight on PBS, pH 7.4. The resulting particles were analyzed for quality and stored at 4°C.

### Nanoparticle characterization with Dynamic Light Scattering

The mean hydrodynamic diameter (z-average), zeta potential, and Polydispersity Index (PI) were measured using a Zetasizer Pro (Malvern Panalytical Ltd). Dynamic Light Scattering using a backscatter measurement was performed in UV cuvettes (Sarstedt).

After dialysis, mRNA concentration on the LNPs was assessed using Quant-iT RiboGreen RNA kit (ThermoFisher), following provider´s instructions.

### Nanoparticle binding/uptake experiments

10^5^ HUVEC (passage 1 to 3), 1,5x10^5^ HUVEC (passage 4 or 5), 3x10^5^ HEK293t cells were plated the day before the experiment. The next day, media was exchanged and liposomes were added at a concentration of 0.5 mM of lipids per well (unless otherwise indicated). After mixing, the plate was incubated for 2h at 37°C (unless otherwise indicated). Then, cells were washed with PBS, detached with trypsin/EDTA and fixed with 4% PFA. Flow cytometry data was acquired on a Cytoflex S (Beckman Coulter). Data analysis and visualization was performed using FlowJo software.

### Calculation of liposome and LNP valency

The number of compound 18 molecules displayed on the outer leaflet of each liposome (ligand valency, κ) was calculated from the liposome diameter, the total lipid concentration, and the molar fraction of DSPE-PEG₂₀₀₀–18 in the formulation, following the framework described by the Protobiology group of Professor Adamala.^[2]^

Briefly, the mean hydrodynamic diameter (d) measured by DLS was used to calculate the surface area of a single liposome (A = 4π(d/2)²). The total number of lipid molecules per liposome was estimated assuming a lipid headgroup area of 0.7 nm² and accounting for both leaflets of the bilayer (N_lipids = 2A / 0.7). The total number of ligand molecules per liposome was then obtained by multiplying N_lipids by the molar fraction of compound 18 in the formulation. Since compound 18 is conjugated via DSPE-PEG₂₀₀₀ and is incorporated into the lipid bilayer, only ligands residing in the outer leaflet are accessible for receptor engagement. Accordingly, the outward-facing valency κ was taken as half the total ligand count per particle (κ = N_ligands / 2). This calculation was performed for each formulation using the experimentally determined particle diameter from DLS and the known mol% of the used ligand.

### Western blotting

Protein extracts were lysed with RIPA buffer (10 mM Tris-HCl pH 8.0, 1 mM EDTA, 1% Triton X-100, 0.1% Sodium Deoxycholate, 0.1% SDS, 140 mM NaCl). 4x Sample buffer was diluted and samples were analyzed by Western blotting on nitrocellulose membranes (Amersham Pharmacia Biotech, UK) using secondary HRP-conjugated antibodies. Proteins were detected by enhanced chemiluminescence (Amersham Life Sciences) on a ChemiDoc MP Imaging System (Biorad).

## Synthetic chemistry

### Instrumentation and general analytical methods.

Mass spectra were obtained on a Shimadzu QP 1000 instrument (EI, 70 eV) and on a Bruker maXis 4G instrument (ESI-TOF, HRMS). ^1^H- and ^13^C-NMR spectra were recorded with a Bruker Avance III 400 spectrometer (400 MHz for ^1^H, 100 or 50 MHz for ^13^C) at 298 K using a directly detecting broadband observe (BBFO) probe. The center of the (residual) solvent signal was used as an internal standard which was related to TMS with δ 7.26 ppm (^1^H in CDCl_3_), 2.49 ppm (^1^H in DMSO-*d*_6_) and δ 77.0 ppm (^13^C in CDCl_3_), 39.5 ppm (^13^C in DMSO-*d*_6_). Data are reported as: s = singlet, d = doublet, t = triplet, q = quartet, p = pentet, m = multiplet, br = broad; coupling constants (*J*) are given in Hz.

Air sensitive reactions were performed under an inert atmosphere of argon using standard Schlenk techniques. Chemicals were purchased from Sigma Aldrich, Acros, ABCR Germany and TCI Europe and were used as received. DSPE-PEG2000-CarboxyNHS was purchased from Avanti Lipid and from NOF Europe. Solutions were evaporated under reduced pressure with a rotary evaporator. For column chromatography, silica gel 60 (0.04-0.063 mm) was used. TLC was carried out on aluminium sheets precoated with silica gel 60 F254 (Macherey-Nagel, Merk); the spots were visualized under UV light (λ = 254 nm) or KMnO_4_ (aq.). Flash chromatography was performed on a Teledyne ISCO Combiflash NextGen 300 system.

### Synthesis, characterization and spectral data of compounds.

2-amino-4-methylpyrimidine-5-carboxylic acid **(9b)**

To a suspension of ethyl-2-amino-4-methylpyrimidine-5-carboxylate **9a** (1.0 g, 5.519 mmol, 1 eq.) in 20 mL EtOH:THF (1:1, v/v) was added a 1N aqueous solution of NaOH (10 mL). The mixture was stirred at 60 °C overnight. 1N HCl was added to adjust the pH to 3. The mixture was concentrated under reduced pressure until a large amount of solid precipitated. The solid was collected by filtration and washed with water and hexane. The product **9b** was obtained as a white solid without any further purification (625 mg, 74%).

**^1^H NMR** (400 MHz, DMSO-*d*_6_) δ: 12.52 (br s, 1H), 8.62 (s, 1H), 7.26 (br s, 2H), 2.51 (s, 3H).

**^13^C NMR** (100 MHz, DMSO- *d*_6_) δ: 169.9, 166.5, 163.8, 161.2, 111.8, 24.25.

**HRMS** (ESI), *m/z*: calcd. for C_6_H_8_N_3_O_2_ 154.0611 [M + H]^+^; found: 154.0608.

benzyl (2-(2-amino-4-methylpyrimidine-5-carboxamido)ethyl)carbamate **(9)**

Compound **9b** (93 mg, 0.0607 mmol, 1 equiv) was dissolved in DMF (10 mL). HATU (254 mg, 0.668 mmol, 1.1 eq.) and DIPEA (0.15 mL, 0.850 mmol, 1.4 eq.) were added and the suspension was stirred at rt for 10 minutes. *N*-Z-Ethylendiamine hydrochloride (126 mg, 0.546 mmol, 0.9 eq.) was added as a solid and the resulting suspension was diluted with another 2 mL of DMF. The reaction was stirred overnight at rt. The reaction was quenched with NaOH 10% and diluted with ethyl acetate. The phases were separated and the organic phase was washed with NaOH 10% and brine. The organic phase was filtered and the product **9** was obtained as a white solid without any further purification (161 mg, 81%).

**^1^H NMR** (400 MHz, DMSO-*d*_6_) δ: 8.28 (s, 1H), 8.19 (t, 1H), 7.37-7.29 (m, 5H), 6.89 (s, 2H), 5.01 (s, 2H), 3.25 (q, *J* = 5.9 Hz, 2H), 3.15 (q, *J* = 6.0 Hz, 2H), 2.35 (s, 3H).

**^13^C NMR** (100 MHz, DMSO-*d*_6_) δ: 166.6, 166.4, 163.0, 157.4, 156.3, 137.2, 128.3, 127.8, 127.7, 118.0, 65.2, 22.7.

**HRMS** (ESI), *m/z*: calcd. for C_16_H_20_N_5_O_3_ 330.1561 [M + H]^+^; found: 330.1561.

*tert*-butyl (2-(2-amino-4-methylpyrimidine-5-carboxamido)ethyl)carbamate **(10a)**

Compound **9b** (250 mg, 1.632 mmol, 1 eq.) was dissolved in dry DMF (15 mL). HATU (683 mg, 1.796 mmol, 1.1 eq.) and DIPEA (0.40 mL, 2.285, 1.4 eq.) were added. After stirring at rt for 10 minutes, a solution of *N*-Boc ethylenediamine (0.34 mL, 1.469, 0.9 eq) in dry DMF (3 mL) was added slowly and the mixture was stirred at rt for 16 hours. The mixture was diluted with ethyl acetate (30 mL) and 10% aqueous NaOH solution (30 mL) was added. The layers were separated, and the organic phase was washed once with 10% aqueous NaOH solution and brine. The organic phase was dried over sodium sulfate and the solvent was removed under reduced pressure to give the desired compound **9b** as a golden solid (475 mg, 98%).

**^1^H NMR** (400 MHz, DMSO-*d*_6_) δ: 8.27 (s, 1H), 8.16 (m, 1H), 3.20 (q, *J* = 6.0 Hz, 2H), 3.07 (q, *J* = 6.0 Hz, 2H), 2.35 (s, 3H), 1.37 (s, 9H).

**^13^C NMR** (100 MHz, DMSO-*d*_6_) δ: 166.6, 166.4, 163.1, 157.4, 155.8, 118.1, 77.7, 28.3, 22.7.

**HRMS** (ESI), *m/z*: calcd. for C_13_H_22_N_5_O_3_ 296.1717 [M + H]^+^; found: 296.1726.

2-amino-*N*-(2-aminoethyl)-4-methylpyrimidine-5-carboxamide **(10)**

Compound **9b** (200 mg, 0.677 mmol, 1 eq) was dissolved in dry methanol (2 mL) and 4M HCl in dioxane (2.65 mL) was added dropwise. After stirring for 16 hours at room temperature, the solvent was removed under reduced pressure to give the compound **9** as a white solid (153 mg, 98%) in the form of a hydrochloride.

**^1^H NMR** (400 MHz, DMSO-*d*_6_) δ: 8.94 (t, J = 5.3 Hz, 1H), 8.78 (s, 1H), 8.32 (m, 3H), 3.48 (q, *J* = 5.8 Hz, 2H), 2.97 (q, *J* = 5.8 Hz, 2H), 2.55 (s, 3H).

**^13^C NMR** (100 MHz, DMSO-*d*_6_) δ: 164.41, 157.46, 117.32, 38.28, 36.96, 22.24.

**HRMS** (ESI), *m/z*: calcd. for C_8_H_14_N_5_O 196.1193 [M + H]^+^; found: 196.1192.

ethyl 2-((2-(((benzyloxy)carbonyl)amino)ethyl)amino)-4-methylpyrimidine-5-carboxylate **(11)**

*N*-Z-ethylenediamine hydrochloride (2415 mg, 10.44 mmol, 3 eq.) was dissolved in dry 1,4-dioxane (45 mL) and ethyl 2-chloro-4-methylpyrimidine-5-carboxylate (700 mg, 3.489, 1 eq.) was added. Triethylamine (4.86 mL, 34.89 mmol, 10 eq.) was added the mixture was refluxed overnight. The solvent was removed under reduced pressure and the residue dissolved in ethyl acetate, washed with brine and dried over sodium sulfate. After evaporation of the solvent under reduced pressure, compound **19** was obtained as a beige solid (1220 mg, 98%).

**^1^H NMR** (400 MHz, CDCl_3_) δ: 8.78 (brs, 1H), 7.32 (m, 5H), 5.95 (brs, 1H), 5.58-5.38 (m, 1H), 5.09 (s, 2H), 4.31 (q, *J* = 7.1 Hz, 2H), 3.61 (m, 2H), 3.44 (m, 2H), 2.63 (s, 3H), 1.36 (t, *J* = 7.1 Hz, 3H).

**^13^C NMR** (100 MHz, CDCl_3_) δ: 165.5, 162.7, 161.4, 159.9, 136.6, 128.6, 128.3, 128.2, 66.9, 60.7, 41.5, 14.5.

**HRMS** (ESI), *m/z*: calcd. for C_18_H_23_N_4_O_4_ 359.1714 [M + H]^+^; found: 359.1715.

2-((2-(((benzyloxy)carbonyl)amino)ethyl)amino)-4-methylpyrimidine-5-carboxylic acid **(12)**

Compound 11 (1.220 g, 3.404 mmol, 1 eq.) was dissolved in methanol (60 mL) and 1N aqueous LiOH (42 mL) was added at 0 °C. The mixture was allowed to reach room temperature overnight. The mixture was diluted with water and extracted three times with dichloromethane at pH = 12. The aqueous layer was acidified to pH = 1 and extracted three times with ethyl acetate. The acidic extracts were combined and dried over sodium sulfate. After evaporation of the solvent under reduced pressure the compound **12** was obtained as a yellow solid (838 mg, 75%).

**^1^H NMR** (400 MHz, DMSO-*d*_6_) δ: 12.53 (brs, 1H), 8.68-8.63 (m, 1H), 7.82-7.76 (m, 1H), 7.37-7.30 (m, 5H), 5.01 (s, 2H), 3.41-3.38 (m, 2H), 3.18 (m, 2H), methyl group signal overlaps with DMSO.

**^13^C NMR** (100 MHz, DMSO-*d*_6_) δ: 169.6, 166.5, 162.3, 162.2, 161.0, 160.9, 156.2, 137.2, 128.3, 127.8, 127.7, 65.2, 24.8, 24.3.

**HRMS** (ESI), *m/z*: calcd. for C_16_H_18_N_4_NaO_4_ 353.1220 [M + Na]^+^; found: 353.1214.

benzyl (2-((5-(isopropylcarbamoyl)-4-methylpyrimidin-2-yl)amino)ethyl)carbamate **(13)**

Compound **12** (390 mg, 1.180 mmol, 1 eq.) was dissolved in dry DMF (10.5 mL). HATU (493 mg, 1.298 mmol, 1.1 eq.) and DIPEA (0.28 mL, 1.652 mmol, 1.4 eq.) were added and the suspension was stirred at room temperature for 10 minutes. A solution of isopropylamine (0.09 mL, 1.062 mmol, 0.9 eq.) in dry DMF (3.5 mL) was added and the resulting suspension was diluted with 4 mL of DMF. The reaction was stirred overnight at rt. The mixture was diluted with ethyl acetate and 10% NaOH was added. The resulting phases were separated and the organic phase was washed with NaOH 10% and brine. The organic phase was dried over sodium sulfate and the solvent was removed under reduced pressure to give compound **12** as a grey solid (331 mg, 76%).

**^1^H NMR** (400 MHz, CDCl_3_) δ: 8.25 (s, 1H), 7.34 (m, 5H), 5.56 (m, 2H), 5.43 (s, 1H), 5.09 (s, 2H), 4.23 (sextet, *J* = 6.9 Hz, 1H), 3.59 (q, *J* = 5.7 Hz, 2H), 3.43 (q, *J* = 5.7 Hz, 2H), 2.50 (s, 3H), 1.60 (s, 2H), 1.25 (d, *J* = 6.9 Hz, 6H).

**^13^C NMR** (100 MHz, CDCl_3_) δ: 167.5, 166.0, 162.1, 156.7, 156.5, 136.5, 128.6, 128.2, 128.1, 119.7, 66.8, 41.9, 41.3, 23.2, 22.8.

**HRMS** (ESI), *m/z*: calcd. for C_19_H_26_N_5_O_3_ 372.2030 [M + H]^+^; found: 372.2026.

2-((2-aminoethyl)amino)-*N*-isopropyl-4-methylpyrimidine-5-carboxamide **(14)**

Compound **13** (324 mg, 0.872 mmol, 1 eq.) and Pd/C 10% (80 mg) were suspended in methanol (10 mL) under inert atmosphere. Hydrogen gas was bubbled in the reaction mixture overnight. The reaction mixture was filtered through celite eluting with methanol. After evaporation of the solvent, compound **15** was obtained as a white solid (198 mg, 96%).

**^1^H NMR** (400 MHz, CDCl_3_) δ: 8.27 (s), 5.58 (m, 2H), 4.22 (sextet, *J* = 6.8 Hz, 1H), 3.51 (q, *J* = 5.8 Hz, 2H), 2.92 (br s, 2H), 2.50 (s, 3H), 1.24 (d, *J* = 6.6 Hz, 6H).

**^13^C NMR** (100 MHz, CDCl_3_) δ: 167.54, 166.34, 162.34, 156.64, 119.42, 44.30, 42.04, 41.62, 23.34, 22.96.

**HRMS** (ESI), *m/z*: calcd. for C_11_H_20_N_5_O 238.1662 [M + H]^+^; found: 238.1666.

benzyl (2-((5-(benzylcarbamoyl)-4-methylpyrimidin-2-yl)amino)ethyl)carbamate *(***15)**

Compound **12** (354 mg, 1.076 mmol, 1 eq.) was dissolved in dry DMF (9 mL). HATU (448 mg, 1.179 mmol, 1.1 eq.) and DIPEA (0.26 mL, 1.500 mmol, 1.4 eq.) were added and the suspension was stirred at rt for 10 min. A solution of Benzylamine (0.10 mL, 0.964 mmol, 0.9 eq) in 4 mL of dry DMF was added to the reaction mixture. The reaction was stirred overnight at rt. The mixture was diluted with ethyl acetate and 10% NaOH was added. The phases were separated, and the organic phase was washed with NaOH 10% and brine. The organic phase was dried over sodium sulfate and the solvent was removed under reduced pressure. Compound **15** was obtained as a white solid (447 mg, 99%) after purification by column chromatography on silica gel (DCM:MeOH, 95:5).

**^1^H NMR** (400 MHz, CDCl_3_) δ: 8.32 (br s, 1H), 7.33 (m, 11H), 5.99 (br s, 1H), 5.59 (br s, 1H), 5.09 (s, 2H), 5.59 (d, *J* = 5.9 Hz, 2H), 3.59 (q, *J* = 5.0 Hz, 2H), 3.43 (q, *J* = 5.0 Hz, 2H), 2.53 (s, 3H).

**^13^C NMR** (100 MHz, CDCl_3_) δ: 168.0, 166.8, 162.3, 156.8, 138.2, 136.6, 129.0, 128.7, 128.3, 128.2, 128.0, 127.8, 119.0, 66.9, 66.0, 44.2, 41.4, 15.4.

**HRMS** (ESI), *m/z*: calcd. for C_23_H_26_N_5_O_3_ 420.2030 [M + H]^+^; found: 420.2035.

2-((2-aminoethyl)amino)-*N*-benzyl-4-methylpyrimidine-5-carboxamide **(16)**

Compound **15** (280 mg, 0.667 mmol, 1 eq.) was dissolved in methanol (10 mL) under inert atmosphere and Pd/C 10% w (70 mg) was added. Hydrogen gas was bubbled in the reaction mixture overnight. The mixture was filtered through celite eluting with methanol. After evaporation of the solvent, compound **16** was obtained as a white solid (136 mg, 72%).

**^1^H NMR** (400 MHz, CDCl_3_) δ: 8.34 (br s, 1H), 7.35 (m, 5H), 6.00 (br s, 1H), 5.60 (br s, 1H), 4.60 (d, *J* = 5.6 Hz, 2H), 3.51 (q, *J* = 5.9 Hz, 2H), 2.92 (t, *J* = 5.9 Hz, 2H), 2.54 (s, 3H).

**^13^C NMR** (100 MHz, CDCl_3_) δ: 167.79, 167.02, 162.27, 156.72, 138.26, 128.85, 127.86, 127.66, 118.59, 77.48, 77.16, 76.84, 44.14, 44.01, 41.44, 23.42.

**HRMS** (ESI), *m/z*: calcd. for C_15_H_20_N_5_O 286.1662 [M + H]^+^; found: 286.1665.

ethyl 2-((2-((*tert*-butoxycarbonyl)amino)ethyl)amino)-4-methylpyrimidine-5-carboxylate **(17)**

Ethyl 2-chloro-4-methylpyrimidine-5-carboxylate (500 mg, 2.492 mmol, 1 eq.) was dissolved in dry 1,4-dioxane (25 mL) and *N*-Boc-ethylenediamine (1.7 mL, 7.476 mmol, 3 eq) was added. Triethylamine (3.47 mL, 24.92 mmol, 3 eq.) was added and the reaction was refluxed overnight. After cooling down to rt, the solvent was removed under reduced pressure. The residue was dissolved in ethyl acetate and washed with brine. After drying over sodium sulfate, the solvent was removed under reduced pressure. The mixture was purified by column chromatography on silica gel (DCM:MeOH, 95:5) to give compound **17** as a white solid (650 mg, 80%).

**^1^H NMR** (400 MHz, CDCl_3_) δ: 8.79 (br s, 1H), 5.93 (br s, 1H), 5.26-5.02 (br, 1H), 4.31 (q, *J* = 7.1 Hz, 2H), 3.60 (q, *J* = 5.6 Hz, 2H), 3.36 (q, *J* = 5.3 Hz, 2H), 2.64 (br s, 3H), 1.42 (s, 9H), 1.37 (t, *J* = 7.1 Hz, 3H).

**^13^C NMR** (50 MHz, CDCl_3_) δ: 165.55, 162.69, 161.37, 156.38, 60.63, 41.75, 28.50, 24.90, 14.46.

**HRMS** (ESI), *m/z*: calcd. for C_15_H_25_N_4_O_4_ 325.1870 [M + H]^+^; found: 325.1876.

2-((2-((*tert*-butoxycarbonyl)amino)ethyl)amino)-4-methylpyrimidine-5-carboxylic acid **(18)**

Compound **17** (140 mg, 0.432 mmol, 1 eq.) was disolved was dissolved in methanol and cooled down to 0 °C. A solution of LiOH (1M in water) was added dropwise and the reaction mixture was stirred overnight at room temperature. The mixture was diluted with water and extracted three times with DCM at pH = 12. The, the aqueous phase was acidified with 2N HCl to reach pH = 1 and extracted three times with DCM. The combined acidic organic extracts were washed with water and dried over sodium sulfate. The solvent was evaporated under reduced pressure. The crude material was used in the next step without purification.

*tert-*butyl (2-((5-(benzylcarbamoyl)-4-methylpyrimidin-2-yl)amino)ethyl)carbamate **(19)**

Compound **18** (167 mg, 0.563 mmol, 1 eq.) was dissolved in dry DMF (6 mL). HATU (235 mg, 0.619 mmol, 1.1 eq.) and DIPEA (0.14 mL, 0.789 mmol, 1.4 eq.) were added and the suspension was stirred at room temperature for 10 minutes. A solution of benzylamine (0.06 mL, 0.506 mmol, 0.9 eq.) in dry DMF (2 ml) was added and the reaction was stirred overnight at rt. The mixture was diluted with ethyl acetate and 10% NaOH was added. The resulting phases were separated, and the organic phase was washed with NaOH 10% and brine. The organic phase was dried over sodium sulfate and the solvent was removed under reduced pressure. Compound **19** was obtained as a brown solid (214 mg, 91%) after purification by column chromatography on silica gel (DCM:MeOH, 9:1).

**^1^H NMR** (400 MHz, CDCl_3_) δ: 8.33 (brs, 1H), 7.36-7.29 (m, 5H), 6.08 (brs, 1H), 5.68 (brs, 1H), 5.09 (brs, 1H), 4.59 (d, *J* = Hz, 2H), 3.60-3.51 (m, 2H), 3.38-3.29 (m, 2H), 2.53 (s, 3H), 1.42 (s, 9H).

**HRMS** (ESI), *m/z*: calcd. for C_20_H_28_N_5_O_3_ 386.2187[M + H]^+^; found: 372.2191.

ethyl 1-(phenylsulfonyl)piperidine-2-carboxylate **(21)**

Ethyl pipecolinate (3.180 mmol, 500 mg, 0.5 ml, 1 eq.) was dissolved in dry DMF (15 ml) and triethylamine (9.540 mmol, 965 mg, 1.33 ml, 3 eq.) was added. Phenylsulfonyl chloride (3.180 mmol, 562 mg, 0.4 ml, 1 eq.) was added dropwise and the reaction was stirred at room temperature for 6 hours. The solvent was removed under reduced pressure. The residue was dissolved in DCM and washed two times with 2M HCl. The organic phase was washed two times with brine, dried over sodium sulfate and the solvent was removed under reduced pressure to give compound **21a** as a yellow oil (930 mg, 98%).

**^1^H NMR (400 MHz, CDCl_3_)** δ**:** 7.79 (m, 2H), 7.55 (m, 1H), 7.47 (m, 2H), 4.74 (d, *J* = 5.5 Hz, 1H), 4.07-3.90 (m, 2H), 3.80-3.76 (m, 1H), 3.23 (dt, *J* = 12.7 Hz, *J* = 3.0 Hz, 1H), 2.16-2.10 (m, 1H), 1.79-1.62 (m, 3H), 1.55-1.43 (m, 1H), 1.33-1.21 (m, 1H), 1.13 (t, *J* = 7.1 Hz, 3H).

**^13^C NMR (50 MHz, CDCl_3_)** δ**:** 170.8, 140.2, 132.5, 128.9, 127.3, 61.2, 55.2, 42.8, 28.0, 24.9, 20.1, 14.2.

**HRMS** (ESI), *m/z*: calcd. for C_14_H_19_NNaO_4_S [M + Na]^+^ 320.0927; found: 320.0949.

1-(phenylsulfonyl)piperidine-2-carboxylic acid **(22)**

Compound **21** (320 mg, 1.076 mmol, 1eq.) was dissolved in methanol (5 mL) and cooled down to 0 °C. A solution of LiOH (1M in water) was added dropwise and the reaction mixture was stirred overnight allowing it to reach room temperature. The mixture was diluted with water and extracted three times with DCM at pH = 12. The, the aqueous phase was acidified with 2N HCl to reach pH = 1 and extracted three times with DCM. The combined acidic organic extracts were washed with water and dried over sodium sulfate. The solvent was evaporated under reduced pressure to give compound **22** as a colorless oil (180 mg, 76% yield).

**^1^H NMR (400 MHz, CDCl_3_)** δ**:** 7.82-7.80 (m, 2H), 7.58-7.54 (m, 1H), 7.50-7.47 (m, 2H), 4.79 (d, *J* = 4.64 Hz, 1H), 3.78-3.75 (m, 1H), 3.24-3.17 (td, *J* = 12.72, 2.87 Hz, 1H), 2.18-2.14 (m, 1H), 1.77-1.63 (m, 3H), 1.50-1.25 (m, 2H).

**^13^C NMR (100 MHz, CDCl_3_)** δ**:** 176.4, 140.1, 132.7, 129.0, 127.2, 54.9, 42.7, 27.6, 24.6, 20.2.

**HRMS** (ESI), *m/z*: calcd. for C_12_H_15_NNaO_4_S [M + Na]^+^ 292.0614; found: 292.0644.

*tert-*butyl (2-(1-(phenylsulfonyl)piperidine-2-carboxamido)ethyl)carbamate **(23)**

Compound **22** (140 mg, 0.520, 1 eq.) and *N*-Boc-ethylenediamine (0.09 mL, 0.623 mmol, 1.2 eq.) were dissolved in dry acetonitrile (5 mL). EDC (100 mg, 0.520 mmol, 1 eq.), HOBt (7 mg, 0.052 mmol, 0.1 eq.), DIMAP (63 mg, 0.520 mmol, ,1 eq.) and DIPEA (0.45 mL, 2.6 mmol, 5 eq.) were added sequentially and the reaction was stirred at room temperature for 24 hours. The solvent was removed under reduced pressure and the residue dissolved in ethyl acetate and washed with saturated aqueous NaHCO_3_ and brine. After drying over sodium sulfate the solvent was removed. The crude mixture was purified by column chromatography on silica gel (hexane:ethylacetate, 1:1) to give compound **23** as a white solid (200 mg, 93%).

**^1^H NMR** (400 MHz, CDCl_3_) δ: 7.85 (m, 2H), 7.62 (m, 1H), 7.55 (m, 2H), 6.97 (brs, 1H), 4.92 (brs, 1H), 4.47 (d, *J* = 5.2 Hz, 1H), 3.91 (m, 1H), 3.45 (m, 1H), 3.28 (m, 3H), 3.06 (m, 1H), 2.22 (m, 1H), 1.43 (s, 9H), 1.39-1.24 (m, 3H), 1.15-1.02 (m, 2H).

**^13^C NMR** (100 MHz, CDCl_3_) δ: 170.3, 140.3, 133.1, 129.5, 127.3, 56.4, 43.7, 40.7, 40.3, 28.5, 24.3, 23.5, 20.0.

**HRMS** (ESI), *m/z*: calcd. for C_19_H_29_N_3_NaO_2_S 434.1720 [M + H]^+^; found: 434.1719

*N*-(2-aminoethyl)-1-(phenylsulfonyl)piperidine-2-carboxamide **(24)**

Compound **22** (200 mg, 0.486 mmol, 1 eq.) was dissolved in dry DCM (4 mL) and 4M HCl in dioxane (1.9 mL) was added dropwise. After stirring for 16 hours at room temperature, the solvent was removed under reduced pressure to give compound **24** as a white solid (140 mg, 92%) (hydrochloride).

**^1^H NMR** (400 MHz, CDCl_3_) δ: 8.18 (brs, 3H), 7.98 (m, 1H), 7.87 (m, 2H), 7.57-7.49 (m, 3H), 4.68 (s, 1H), 3.78-3.69 (m, 3H), 3.31-3.25 (m, 3H), 2.14 (m, 1H), 1.42-1.21 (m, 5H)

**^13^C NMR** (100 MHz, CDCl_3_) δ: 171.76, 140.21, 132.96, 129.41, 127.41, 56.47, 43.85, 40.22, 37.66, 25.75, 23.63, 19.89.

**HRMS** (ESI), *m/z*: calcd. for C_14_H_22_N_3_O_3_S 312.1376 [M + H]^+^; found: 312.1378.

### Synthesis schemes

### 1H and 13C NMR spectra

# Supporting figures

| **%mol Compound 18** | **Size (nm)** | **PI** |
| --- | --- | --- |
|  |  |  |
| 0 | 94.36 | 0.2104 |
| 0.4 | 82.70 | 0.2113 |
| 0.6 | 89.88 | 0.2200 |
| 0.8 | 94.05 | 0.2034 |
| 1 | 83.26 | 0.1890 |
| 2 | 90.78 | 0.1805 |

mol% **18**:

b)

a)

### Figure S1. Size characterization of compound 18–decorated liposomes.

(a) Intensity-weighted hydrodynamic size distributions measured by DLS for PEGylated liposomes containing 0–2 mol% compound 18 (black to dark-green traces as indicated). All formulations show a single, narrow population with mean diameters in the 80–95 nm range. (b) Table summarizing Z-average diameters and polydispersity indices (PDI); incorporation of up to 2 mol% compound 18 maintains sizes of 83–94 nm with low polydispersity (PDI 0.18–0.22).

### Figure S2. Uptake experiment in HEK293T cells using 18 decorated liposomes show no increase over NT control

**a**) Effect of ligand valency on internalization. HEK293T WT cells treated with liposomes displaying increasing valencies of 18 (35–700 ligands per liposome) show no valency-dependent increase in MFI. **b**) Time-course of cellular uptake. HEK293T WT treated with NT (grey) or 18-decorated liposomes (green) (0.5 mM) were analyzed at 2 h, 6 h and 24 h and show no time dependent accumulation within the tested intervals. **c**) Dose-response analysis. Uptake of NT (grey) and 18-decorated (green) liposomes at increasing lipid concentrations (0.25–1.0 mM) show no increase in MFI values for targeted liposomes over untargeted. Data represent mean ± SD (n = 4), Statistical significance was assessed by two-way ANOVA with Dunnett's multiple comparisons test against the NT control (a), or two-way ANOVA with Sidak's multiple comparisons test (b, c), ns= non-significant

### Figure S3. Rapid endocytosis of integrin αVβ3 on pHUVECs

Flow-cytometric analysis of surface integrin αVβ3 on pHUVECs stained with a FITC-conjugated anti-αVβ3 antibody and chased at 37 °C for the indicated times. Mean fluorescence intensity (MFI) decreases markedly over 30 min, consistent with rapid endocytosis of surface αVβ3. Bars on the right show staining with a FITC-labeled isotype control antibody at 4 °C and 37 °C, indicating that the temperature-dependent signal loss is specific for αVβ3 and not due to nonspecific effects on antibody binding.

### Figure S4. Donor-dependent integrin αVβ3 expression in pHUVECs

Flow-cytometric histograms of surface integrin αVβ3 on pHUVECs isolated from 5 different donors, stained with an anti-αVβ3 antibody (colored traces) or isotype IgG control (lighter traces). Donors 1–3 show comparable αVβ3 levels, whereas donor 4 exhibits higher expression at early passage that decreases in almost-senescent cells; donor 5 displays intermediate expression. In all cases, antibody-stained populations are clearly shifted relative to IgG controls, confirming specific αVβ3 expression across donors.

| **%mol cRGDfK** | **Size (nm)** | **PDI** |
| --- | --- | --- |
| 0 | 83.49 | 0.1195 |
| 0.01 | 89.71 | 0.175 |
| 0.05 | 81.23 | 0.2121 |
| 0.1 | 86.75 | 0.2401 |
| 0.5 | 78.01 | 0.2076 |
| 1 | 79.68 | 0.1806 |
| 2 | 65.27 | 0.1751 |

b)

a)

### Figure S5. Size characterization of cRGDfK–decorated liposomes.

(a) Intensity-weighted hydrodynamic size distributions measured by DLS for PEGylated liposomes containing 0–2 mol% cRGDfK (color gradient as indicated). All formulations show a single, narrow population with mean diameters in the 65–90 nm range. (b) Table summarizing Z-average diameters and polydispersity indices (PDI); incorporation of up to 2 mol% cRGDfK maintains small liposome sizes (65–90 nm) with low polydispersity (PDI 0.12–0.24).

### Figure S6. Soluble cRGDfK competes with cRGDfK–decorated liposome binding.

Flow-cytometric analysis of integrin αVβ3-expressing cells incubated with PBS, naked liposomes, or liposomes displaying 0.5 or 2 mol% cRGDfK in the absence (–) or presence (+) of 0.5 mM free cRGDfK. cRGDfK-liposomes show markedly increased mean fluorescence intensity (MFI) compared to PBS and naked liposomes, whereas addition of excess soluble cRGDfK strongly reduces binding, consistent with competitive, RGD-mediated recognition. Data represent mean ± SD (n = 3). Statistical significance was assessed by one-way ANOVA with Tukey's multiple comparisons test, *: p<0.05 ,***: p<0.001.

a)

b)

| LNPs | Size (nm) | PDI |
| --- | --- | --- |
| NT | 116.5 | 0.04496 |
| 0.45 mol% 18 | 154.0 | 0.07939 |

### Figure S7. Size characterization of compound 18–decorated LNPs.

(a) Intensity-weighted hydrodynamic size distributions measured by DLS for MC3-based LNPs without ligand (black) or containing 1.5 mol% compound 18 (green). Both formulations show a single, narrow population, with a moderate size increase upon ligand incorporation. (b) Table summarizing Z-average diameters and polydispersity indices (PDI); NT LNPs and 1.5 mol% 20–LNPs display mean diameters of 117 and 154 nm, respectively, with low polydispersity (PDI ≤ 0.08).

### Figure S8. Compound 2/25 series with similar biophysical affinity and targeting performance in pHUVECs fails to increase mRNA translation.

a) NHS coupling of 24 to derive DSPE-PEG coupled ligand for targeting (25). b) Time-course of cellular uptake. pHUVECs treated with NT or 25-decorated liposomes (0.5 mM) were analyzed at 30 min, 2 h, and 6 h, confirming progressive accumulation in line with fast rapid receptor cycling. c) Evaluation of valency dependance. pHUVECS were incubated for 2 hours with NT or targeted liposomes displaying 130-1300 copies of 25 on their surface. d) Concentration-dependent uptake. pHUVECs were incubated with NT (NT, black) or 25-decorated (green) liposomes (0–1.5 mM lipid concentration). e) 25 decorated LNPs fail to mediate functional cytosolic delivery and translation of eGFP mRNA in pHUVECs. Cells were treated with NT (NT, grey) or 18-decorated (green) LNPs encapsulating 2, 5 or 7µg/ml eGFP mRNA. f) No competition is observed between 8/18 and 2/25 series. Uptake experiments of targeted liposomes in pHUVECs show no cross competition between free or liposomal ligands. Statistical significance was assessed by two-way ANOVA with Sidak's multiple comparisons test (b, d, e, f) and Dunnett's multiple comparisons test against the NT control (c). Data represent mean ± SD (n = 4), *: p<0.05 ,***: p<0.001,****: p<0.0001 . ns: not significant. g) waveRAPID sensorgram of 2 shows similar kD to 8 (Fig. 2c).

# Supporting references

[1]	J. Aretz, E.-C. Wamhoff, J. Hanske, D. Heymann, C. Rademacher, “Computational and Experimental Prediction of Human C-Type Lectin Receptor Druggability” *Front. Immunol.* **2014**, *5*, DOI 10.3389/fimmu.2014.00323.

[2],	“Liposome calculation – protobiology.org,” can be found under http://protobiology.org/wp3/2021/03/17/liposomecalculation/(accessed 30 March 2026), **2021**.
